# Ferroptosis in tumor immunity and therapy

**DOI:** 10.1111/jcmm.17529

**Published:** 2022-11-01

**Authors:** Chuandong Gong, Qiankun Ji, Miaojing Wu, Zewei Tu, Kunjian Lei, Min Luo, Junzhe Liu, Li Lin, Kuangxun Li, Jingying Li, Kai Huang, Xingen Zhu

**Affiliations:** ^1^ Department of Neurosurgery The Second Affiliated Hospital of Nanchang University Nanchang China; ^2^ Institute of Neuroscience, Nanchang University Nanchang China; ^3^ Jiangxi Key Laboratory of Neurological Tumors and Cerebrovascular Diseases Nanchang China; ^4^ College of Queen Mary Nanchang University Nanchang China; ^5^ Department of Comprehensive Intensive Care Unit Second Affiliated Hospital of Nanchang University Nanchang China

**Keywords:** ferroptosis, immune, lipid peroxidation, therapy, tumor microenvironment

## Abstract

Ferroptosis is a type of regulated cell death (RCD), and it plays an important role in the occurrence of diseases, especially the development of tumors. Ferroptosis of tumor cells affects the antitumor immunity and the immune response to treatment to varying degrees. Ferroptosis also plays a key role in immune cells. This review outlines the mechanism of the immune‐related effects of ferroptosis pathways in tumor progression and treatment, and it discusses potential methods for improving antitumor immunity and enhancing the efficacy of current cancer treatments by targeting ferroptosis.

## 1 INTRODUCTION

Ferroptosis is a newly discovered oxidative cell death triggered by excessive iron‐mediated lipid peroxidation.^1^, ^2^ It was first reported in 2012 when it was found that some small molecules (e.g., erastin and RSL3) could specifically induce a non‐apoptotic form of RCD in cancer cells; this RCD could be blocked by lipophilic antioxidants or iron chelators (e.g., vitamin E and ferrostatin‐1).^3^ The morphological characteristics of ferroptotic cells are different from those of apoptotic cells. Apoptotic cells are characterized by cellular shrinkage, membrane blebbing, chromatin condensation, and nuclear fragmentation, whereas ferroptotic cells show typical necrotic features, such as an incomplete plasma membrane and the release of intracellular contents, especially damage‐associated molecular patterns (DAMPs).^4^ Ultrastructural analysis has also shown that the mitochondria of apoptotic cells become larger,^5^ whereas the mitochondria of ferroptotic cells lose their structural integrity and become smaller in size, the mitochondrial bilayer membrane density increases, cristae decrease or disappear, and the outer membrane ruptures. In addition, ferroptotic cells had normal‐sized nuclei without chromatin aggregation.^3^

Here, we outline the role of the ferroptosis pathway in the tumor microenvironment (TME). Ferroptosis of tumor cells and immune cells in the TME is regulated by various factors, thereby inhibiting or promoting tumor progression. The impact of ferroptosis on tumors may be environmentally dependent. Changes in tumor genetics and staging, as well as changes in host factors, will alter the role of ferroptosis. The immune system not only plays a vital role in preventing the occurrence, development, and metastasis of tumors but also determines the response of tumors to treatment. Tumor cells can evade the surveillance of the immune system in a variety of ways, such as reducing immunogenicity, directly suppressing the immune response, and promoting the formation of an immunosuppressive network.^6^ Recently, immunotherapy has promoted a successful antitumor immune response that combats tumors by activating the immune system. One of the most striking examples is immune checkpoint blockade therapy, which can reactivate the ability of T cells to kill tumor cells.^7^, ^8^ In addition, other immunotherapies, including cytokine therapy, dendritic cell vaccines, and chimeric antigen receptor T cells, can promote antitumor immunity.

Recent studies have found that the ferroptosis pathway is involved in the survival, apoptosis, differentiation, activation, and effector functions of various immune cells.^9^, ^10^, ^11^ Moreover, tumor ferroptosis can regulate tumor growth by modulating the immune response. By combining immune checkpoint therapy with ferroptosis inducers, tumor resistance can be eliminated. As understanding about the multi‐layered relationship between ferroptosis pathways and tumor immune responses has progressed, and as the potential to target ferroptosis pathways in cancer treatment has developed, it has become appropriate to summarize and discuss the latest findings about ferroptosis pathways in tumor immunity and immunotherapy.

## 2 MECHANISM OF FERROPTOSIS

### 2.1 Inhibition of system Xc‐

System Xc‐, a heterodimeric cell surface antiporter for amino acids, contains two integral proteins: one is a twelve‐pass transmembrane transporter protein named SLC7A11, and the other is a single‐pass transmembrane regulatory protein named SLC3A2 that is linked with SLC7A11 by a disulfide bridge.^12^ By controlling the process of bidirectional transmembrane transfer of extracellular cystine and intracellular glutamic acid, System Xc‐ improves the concentration of intracellular cystine.^13^ Cystine acts as a necessary substrate for biosynthesis of glutathione (GSH), which is an essential antioxidant that prevents damage to important cellular components.^14^ Likewise, intracellular cystine depletion occurs when system Xc‐ is inhibited. Several chemical molecules and their derivatives can inhibit system Xc‐. For instance, the classical oncogenic molecule, erastin—also known as the first compound discovered to induce ferroptosis—achieves its function by inhibiting system Xc‐.^3^

### 2.2 The role of GPX4 in ferroptosis

**FIGURE 1:**
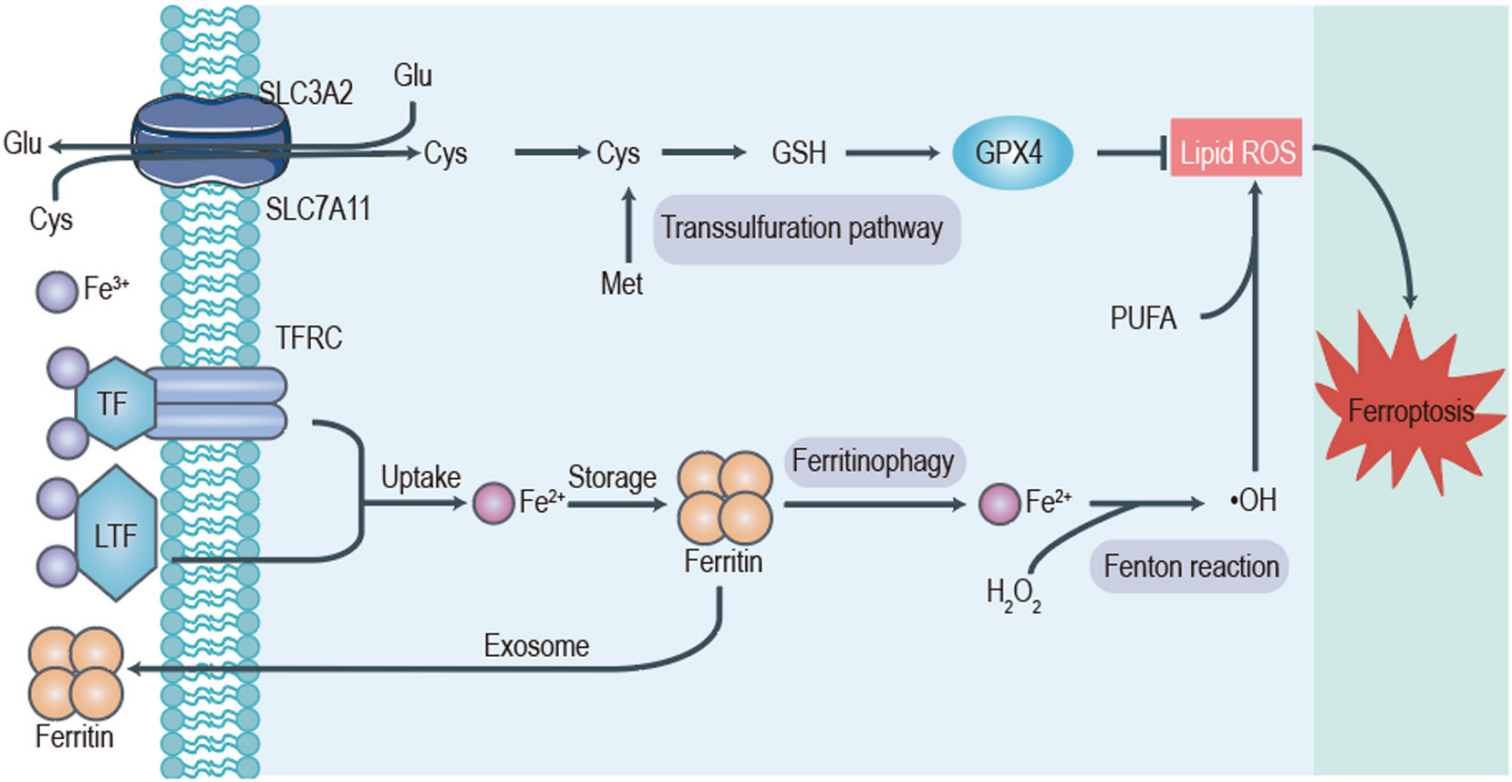
Mechanism of ferroptosis. The mechanism of ferroptosis involves two core parts: one is the xc‐GSH‐GPX4 system; the other is iron metabolism. System Xc‐ is composed of SLC7A11 and SLC3A2; it is responsible for transporting extracellular cystine into the cell and transporting intracellular glutamate out of the cell. Cystine is subsequently involved in the synthesis of GSH, an important antioxidant substance in cells that can inhibit ferroptosis. In addition, transferrin (TF) and lactotransferrin (LTF) import iron into cells. Excessive free Fe^2+^ induces the production of lipid reactive oxygen species (ROS) through the Fenton reaction and ultimately promotes the occurrence of ferroptosis in the cell.

As a GSH‐dependent enzyme, GPX4 can decrease lipid hydroperoxide (LOOH) to harmless alcohol (LOH). Specifically, GPX4 restricts the biosynthesis of highly reactive lipid alkoxy radicals.^15^ Appropriate functioning of GPX4 seems essential for cell survival because GPX4 can efficiently remove phospholipid hydroperoxides. If GPX4 does not effectively clear excessive phospholipid hydroperoxides in cells, an iron‐involved catalytic reaction will be eventually triggered, thus causing ferroptosis.^16^ When GPX4 activity is suppressed, reactive oxygen species (ROS) quickly accumulate in cells and mediate a continual cell damage, exposing cells to a high risk of death (Figure 1). The loss of GPX4 also appears lethal to murine embryos.^15^, ^17^ Inactivation of GPX4 is a key process to cause ferroptosis, and this process can be mediated by RSL3, a small molecule; overexpression of GPX4 can block its function. Ferroptosis can be induced in human oncogenic HRAS cells by knocking down GPX4.^17^ In addition, lipophilic antioxidants and iron chelators can suppress ferroptotic cell death induced by GPX4 deletion in murine cells, which indicates that GPX4 activity is necessary to prevent ferroptosis.^15^, ^18^ One study showed that GPX4 activity plays an important role in several cancers driven by oncogenic mutations and dedifferentiated states.^19^ However, GPX4 is not limited to one mode of cell death. Some studies have reported that the link between GPX4 activity and sensitivity regulates cell death pathways, such as apoptosis,^20^ necrosis,^21^ and pyrolysis.^22^ However, whether these cell death pathways really have similar metabolic characteristics remains to be explored.^23^

### 2.3 The effect of iron in ferroptosis

Iron is a necessary participant in ferroptosis. Cell ferroptosis induced by erastin, RSL3, or a physiological stimulus (e.g., high concentrations of extracellular glutamate) is less likely to occur when iron chelators are added into the growth medium. In addition, both membrane‐permeable (e.g., ciclopirox, and 2,2‐bipyridyl) and membrane‐impermeable (e.g., deferoxamine) iron chelators have been effective in preventing ferroptosis.^3^, ^17^ Moreover, when TFRC (a gene that encodes the transferrin receptor, which is needed to absorb transferrin‐iron complexes) was silenced, erastin and cystine deprivation failed to cause ferroptosis.^24^ Conversely, when the growth medium was supplemented with divalent iron (e.g., ferric ammonium citrate) or iron‐bound transferrin, erastin‐induced ferroptosis was augmented.^3^, ^24^ These results strongly support the essential role of iron in ferroptosis (Figure 1).

Iron chelators may block ferroptosis by keeping iron from offering electrons to oxygen—an offering that is necessary for ROS—but several puzzles remain before a redox‐independent role of iron in ferroptosis is clear.^25^ A study of the properties of iron chelators showed that lipophilic iron chelators pass through the cell membrane, thus reducing the level of free intracellular iron from a “redox‐active” iron pool.^26^ This iron pool provides the ability to catalyze the synthesis of soluble radicals, which can start or accelerate oxidative polyunsaturated fatty acid (PUFA) fragmentation; after ROS synthesis is inhibited, cell death may be blocked.^27^ If iron metabolism is out of control, ROS can be generated, and this process is the most obvious mechanism for ferroptosis.^1^

With the help of transferrin (TF) and transferrin receptor 1 (TFR1), circulated iron (Fe^3+^) can be absorbed into cells; then, an iron oxide reductase named six‐transmembrane epithelial antigen of the prostate 3 deoxidizes Fe^3+^ to Fe^2+^. Iron will eventually release into the labile iron pool (LIP). If the LIP continues to be formed, the process of ROS generation mediated by the interaction between Fe^2+^ and hydrogen peroxide (H_2_O_2_), also known as the Fenton reaction, may develop as a result of the high solubility of Fe^2+^ and its ability to transfer electrons.^28^ Unlike RAS‐unmutated ferroptosis‐insensitive cells, RAS‐mutated ferroptosis‐sensitive cells express a higher level of TFR1 and a lower level of ferritin light chain and ferritin heavy chain 1, which are iron‐storage proteins. When cells continually absorb iron as the intracellular iron storage capacity decreases, iron overload develops and eventually leads to ferroptosis.^29^ Therefore, maintaining homeostasis of iron metabolism in cells is vital for occurrence and progression of ferroptosis; compared with normal cells, cancer cells are more likely to have iron toxicosis and ROS accumulation because of a strong iron dependency. This distinguishing feature may be applied in cancer treatments by inducing ferroptosis in cancer cells.^30^, ^31^

Some additional mechanistic pathways are worthy of discussion. Nuclear factor (erythroid‐derived 2) like 2 (Nrf2) can regulate cellular iron metabolism, and Nrf2 activity has inhibited ferroptosis in hepatocellular carcinoma cells.^32^ The classical tumor suppressor P53 is an important regulator of the ferroptosis process as well. Furthermore, analyses of ferroptosis‐related molecules have identified various ways to regulate the process of ferroptosis in vitro and in vivo.

### 2.4 Lipid oxidation: Result of ferroptosis

When system Xc‐ is inhibited or GPX4 is inactivated, cellular iron‐dependent ROS accumulate and PUFAs are exhausted, ultimately leading to ferroptosis.^17^, ^18^, ^33^ ROS are usually synthesized using the PUFA chains from membrane lipids. PUFAs are easily oxidized either through an enzymatic (e.g., lipoxygenase‐catalyzed) or a non‐enzymatic (e.g., ROS‐catalyzed) method, and this oxidation contributes to the occurrence of LOOH.^27^ LOOH can be transformed into several harmful lipid radicals, such as the alkoxy radical LOH. These lipid radicals can steal protons from nearby PUFAs, thus starting a new wave of lipid oxidation, causing damage to the cellular lipid membrane.^27^

Ultimately, PUFAs become fragmented after oxidation and damage by free toxic radicals.^27^ Ferritin 1 (a kind of small‐molecule antioxidant) can prevent murine cancer cell ferroptosis induced by erastin or GPX4 inactivation from an L‐ROS (lipid ROS) stockpile, increasing PUFA exhaustion and, eventually, ferroptotic cell death; this finding indicates that lipid ROS‐ mediated damage is necessary for ferroptosis^17^, ^18^, ^33^ (Figure 1). The complicated interplay of iron, cysteine, and lipid metabolism maintains an important role in ferroptosis.

## 3 FERROPTOTIC CANCER CELLS IN THE IMMUNE RESPONSE

### 3.1 Immune cell activation

Previous studies have confirmed that ferroptosis belongs to a type of immunogenic cell death (ICD) process^4^, ^34^, ^35^ and therefore has some characteristics of immunogenic cell death. Cells undergoing typical ICD can release DAMPs, such as HMGB1(high‐mobility group protein box 1), adenosine triphosphate (ATP), and calreticulin (CRT). These DAMPs may interact with pattern recognition receptors, phagocytosis receptors, (receptor for advanced glycation endproducts) RAGE, toll‐like receptor 2 (TLR2), toll‐like receptor 4 (TLR4), and purinergic receptors on immune cells to promote their immune response and adaptive immunity driven by cytotoxic T lymphocytes.^36^, ^37^, ^38^ Therefore, research on the immunogenicity of ferroptotic tumor cells and the exploration of its influence on the effect of antitumor treatments have important scientific and clinical significance.

**FIGURE 2:**
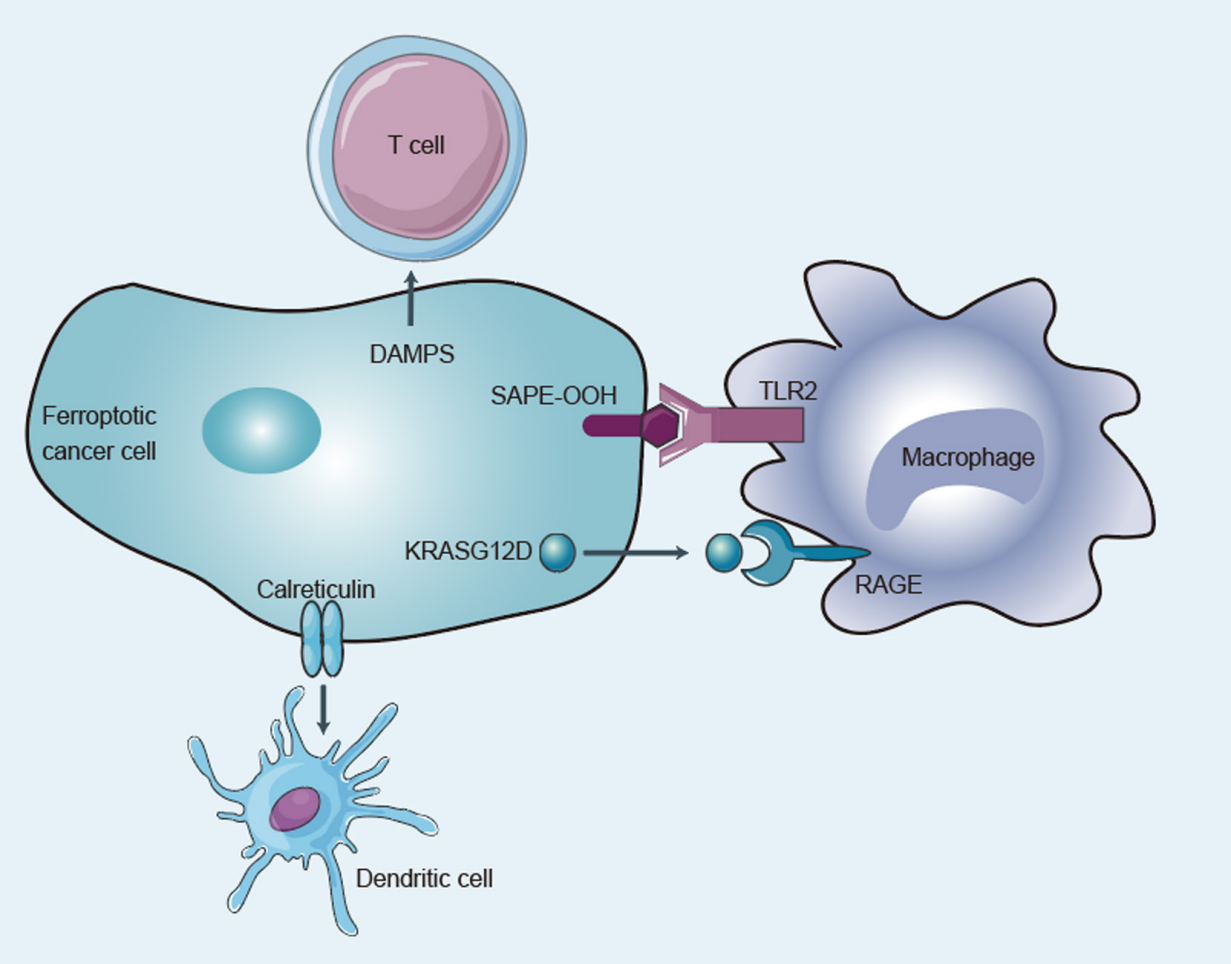
Effect of ferroptotic cancer cells in the tumor microenvironment. Ferroptotic cancer cells can affect immune cells in the tumor microenvironment in many ways to promote or inhibit the antitumor immune response. Various damage‐associated molecular patterns (DAMPs) produced by ferroptotic cancer cells can promote the differentiation and maturation of T cells and dendritic cells to enhance their immune functions. In addition, 1‐steaoryl‐2‐15‐HpETE‐sn‐glycero‐3‐phosphatidylethanolamine (SAPE‐OOH) on the surface can promote the phagocytosis of macrophages through toll‐like receptor 2 (TLR2). Conversely, ferroptotic cancer cells can secrete a large amount of KRAS^G12D^, which promotes the polarization of M1 macrophages into M2 macrophages and damages their ability to kill tumor cells.

Wen et al.^4^ found that HMGB1 released by ferroptotic cells could lead to upregulation of tumor necrosis factor (TNF) expression in macrophages. An increase in HMGB1 and the infiltration of leukocytes were also observed in the ferroptotic tissue of a murine model of experimental pancreatitis.^39^ Interleukin‐33 is a DAMP with high pro‐inflammatory activity.^40^ One study has shown not only that interleukin‐33 in the kidney and plasma increased in a murine model of acute kidney injury with ferroptosis but also that a ferroptosis inhibitor could inhibit this increase.^41^ Another study confirmed that 1‐steaoryl‐2‐15‐HpETE‐sn‐glycero‐3‐phosphatidylethanolamine (SAPE‐OOH) is an “eat me” signal on the surface of ferroptotic cell membranes that increased the phagocytic function of macrophages by targeting TLR2^42^ (Figure 2).

Calreticulin is a soluble endoplasmic reticulum–related chaperone protein on the plasma membrane that is released when cells are stressed or die. It can eliminate cancer cells by promoting the phagocytosis of phagocytes.^43^, ^44^, ^45^, ^46^, ^47^ In ROS‐mediated ferroptotic cancer cells, calreticulin is transferred to the cell surface.^48^, ^49^ Together, these results underscore the idea that ferroptotic cells are able to release signals that let immune cells locate them (Figure 2).

Efimova et al.^34^ established a preclinical model to prove that RSL3, a GPX4 inhibitor, induced the death of murine fibrosarcoma MCA205 cells through ferroptosis rather than *via* apoptosis or necrosis. The authors compared the effects of early and late ferroptotic cancer cells (MCA205 cells treated with RSL3 for 1 h and 24 h, respectively) on the maturation of murine bone marrow–derived dendritic cells (BMDCs) and concluded that the former released HMGB1 and that ATP contributed to the maturation of BMDCs, whereas the latter did not. This finding is the first confirmation that ferroptosis is indeed a kind of ICD.^34^ In addition, ferroptosis induced by photodynamic therapy (PDT) promoted the release of HMGB1/ATP from cancer cells and the subsequent phagocytosis by macrophages and activation and maturation of BMDCs.^50^ These studies have expanded our understanding of the relationship between ferroptosis and ICD and have consolidated the effect of ICD induction to promote immunity after ferroptosis is induced.

In addition to relying on the secretion of annexin A1, the release of type‐1 interferons, and the surface exposure of calreticulin—methods that also apply to apoptotic ICD—ferroptotic^36^ ICD may depend on other DAMPs. The possibility that certain combinations of DAMPs that have not been defined so far may play a role in different types of ICDs has been proposed. Therefore, additional research is urgently needed to support or disprove the hypothesis that different cell death patterns, such as apoptosis, ferroptosis, and necrosis, may cause ICD *via* different approaches despite sharing only a small portion of DAMPs.^51^

Previous research has revealed that apoptotic cells can produce a series of signals during their death, including “find me” and “eat me” signals, which help communicate with immune cells.^52^ Recent studies have demonstrated that ferroptotic cells also produce a corresponding “find me” signal that involves lipid mediators and is able to help immune cells find dead or dying ferroptotic cells. Ferroptotic cancer cells *in vitro* can be located and engulfed by macrophages efficiently with the help of such signals.^53^ Additionally, ferroptotic cells can secrete several oxidative products of AA (arachidonic acid) as the potential signals to regulate antitumor immunity and of lipid mediators. Accordingly, as a ferroptotic signal, LOX (lipoxygenase) not only plays a key role in the oxidation of esterified PUFAs but also promotes the release of such signals from ferroptotic cells, thereby regulating antitumor immunity. Specifically, ferroptotic cells can secrete eicosanoids, including 5‐HETE, 11‐HETE, and 15‐HETE, when GPX4 is depleted.^18^ In cells stimulated by TNF or IL‐1, increased GPX4 activity suppresses the synthesis of pro‐inflammatory lipid mediators, including LTB4, that plays an important role in tumorigenesis and reduces pro‐inflammatory activity activated by the nuclear factor‐kappa B pathway.^54^, ^55^

Esterified eicosanoids are a family of molecules formed under the direct catalyzation of lipoxygenase on SN‐2 fatty acids or by re‐esterification of free eicosanoids. There is a growing interest in their biological role and speculation that they may have the same role as that of immunoregulatory signaling molecules as free eicosanoids.^56^ Analyses of lipid components in the ferroptotic cells have confirmed the presence of a large number of doubly and triply oxidized arachidonic acids, including albumin phosphatidylethanolamine (PE), which may be formed under catalyzation by arachidonic acid 15‐lipoxygenase (ALOX15).^57^ Previous research has described the function of lyso‐phospholipids (a kind of hydrolysis product of PE) in APC attraction to apoptotic cells.^58^ Furthermore, evidence has confirmed that by providing more AA, cancer cells undergoing ferroptosis have accelerated the biosynthesis of eicosanoids, which eventually promoted antitumor immunity.^59^

In conclusion, ferroptosis of tumor cells appears to not only lead to tumor cell death but also promote tumor antigen presentation, immune cell localization, and activation of effector immune cell function in the TME.

### 3.2 Immune response inhibition

Several molecules secreted from ferroptotic cells may promote tumor progression by suppressing immune cells. Cells undergoing ferroptosis can release molecules, including the mutated KRAS^G12D^ protein, that promote tumor development.^60^, ^61^ When the KRAS^G12D^ protein is released and absorbed by macrophages *via a* specific cell surface receptor called RAGE (an advanced glycation end‐product), it induces fatty acid oxidation and promotes the conversion of macrophages into M2‐like tumor‐associated macrophages (TAMs) and activates the tumor‐promoting effect of macrophages. In fact, the expression level of KRAS^G12D^ in TAMs has been positively related to poor prognosis of pancreatic cancer, suggesting that KRAS^G12D^ may be a target for tumor immunotherapy^60^ (Figure 2).

A recent study demonstrated that stearoyl‐CoA desaturase‐1 (SCD1) and fatty acid binding protein (FABP4) play roles in tumor recurrence by reducing the sensitivity of cancer cells to ferroptosis. Specifically, SCD1 secreted by cancer cells increased the production of monounsaturated fatty acids (MUFAs). FABP4 increased the synthesis of lipid droplets under hypoxic circumstances, which both promoted tumor migration and protected cancer cells from ferroptosis.^62^

Ferroptotic cancer cells can generate oxidized lipids, such as 5‐hydroxyeicosatetraenoic acid (5‐HETE), that suppress immune cells to modulate antitumor immunity.^18^ The level of GPX4 activity has been negatively related to the production of pro‐inflammatory lipids, such as leukotriene B4 (LTB4), that are essential for tumorigenesis.^9^, ^54^ Free eicosanoids and esterified eicosane also regulate the immune response. Specifically, oxidized phosphatidylcholine suppressed the maturation of dendritic cells by activating Nrf2 and the differentiation of T‐helper 17 cells.^63^ Moreover, oxidized lipids and lipid droplets also modulate antitumor immune reactions, which can be decomposed to oxygenated neutral lipids, free PUFAs, or PUFA triacylglycerols, that could trigger inaccurate antigen cross presentation and incomplete antitumor immunity.^64^, ^65^ All these lipids suppress antitumor immunity through various mechanisms.

Recent studies have found several links between ferroptotic tumor cells and increased production of prostaglandin E2 (PGE2), which is an important immune modulator that inhibits antitumor immunity.^17^, ^66^, ^67^ PGE2 suppresses the accumulation of classical type 1 dendritic cells and the secretion of CC chemokine ligand 5 (CCL5) and chemokine lymphotactin by natural killer (NK) cells.^67^, ^68^, ^69^ Moreover, PGE2 can also affect an acquired immune response by directly suppressing the function of cytotoxic T cells.^70^ A study by Kurtova et al.^71^ found that the release of PGE2 during chemotherapy was associated with the resistance of a tumor to treatment, and its mechanism was promotion of tumor cell regeneration, which could be blocked by co‐targeting PGE2. Therefore, researchers assumed that tumor cells undergoing ferroptosis are likely to secrete more PGE2 because of the inactivation of GPX4, whereas tumor cells treated with chemotherapy would produce more PGE2, that in turn would promote the growth of ferroptosis‐sensitive tumor cells. Such assumptions lead to the question of how to keep the balance between antitumor immune reactions induced by ferroptosis and the immunosuppressive effect of PGE2 which was promoted by ferroptosis‐sensitive cells.^56^, ^72^, ^73^

One bold hypothesis states that ferroptotic cells in a tumor may inhibit the immune response and promote tumor growth. However, this hypothesis requires more in‐depth research to validate the concept and determine the immunoregulatory role of ferroptosis‐sensitive cells in antitumor immunity. This research may include the analysis of eicosanoids (as immunosuppressive factors) during different levels of GPX4 activity.^18^

## 4 FERROPTOSIS IN TUMOR‐INFILTRATING IMMUNE CELLS

### 4.1 Effector T‐cell subsets

Studies have shown that the increased expression of CD36 in tumor‐infiltrating CD8^+^ T cells in the TME led to tumor progression and poor survival rates in human and mouse cancers. However, genetic downregulation of CD36 in effector CD8^+^ T cells promoted the production of cytotoxic cytokines and eradication of tumors.^74^ In addition, lipid peroxidation and ferroptosis induced by CD36 inhibited the production of cytotoxic cytokines in tumor‐infiltrating CD8^+^ T cells. Therefore, blocking CD36 or inhibiting ferroptosis in CD8^+^ T cells can effectively restore their antitumor functions; more importantly, binding to anti–PD‐1 antibodies had a stronger antitumor effect.^75^

Overexpression of GPX4 or the ferroptotic inhibitor 1 (FSP1) has protected CD8^+^ T cells from ferroptosis without impairing their function. The genetic deletion of ACSL4 could also inhibit the ferroptosis of CD8^+^ T cells but impairs its antitumor function.^76^ A study has shown that conditional deletion of GPX4 induced T‐cell ferroptosis in mice and led to a lack of immune response to infection.^9^ This finding suggests that GPX4 and ferroptosis play important roles in the immune response mediated by T cells.

### 4.2 Myeloid‐derived suppressor cells

Myeloid‐derived suppressor cells (MDSCs) are a type of immunosuppressive cells that accumulate in large amounts under pathological conditions to suppress T‐cell immune responses.^77^ Neutral ceramidase (N‐ceramide hydrolase) is overexpressed in tumor‐infiltrating bone marrow mesenchymal stem cells of colon cancer as an MDSC survival factor. ASAH2 inhibits the p53 pathway of bone marrow mesenchymal stem cells in the TME by destroying the stability of the p53 protein, thereby protecting bone marrow mesenchymal stem cells from ferroptosis. As an inhibitor of ASAH2, NC06 can reduce GSH synthesis and increase lipid ROS to activate ferroptosis in bone marrow mesenchymal stem cells and promote the activation of tumor‐infiltrating cytotoxic T lymphocyte (CTL) to inhibit tumor growth *in vivo*.^78^ Therefore, targeting ASAH2 with NC06 to induce MDSC ferroptosis may be a potentially effective approach to tumor immunotherapy.

### 4.3 Macrophages

In the process of cancer therapy, eliminating dead cells *via* macrophages is very important in maintaining immune homeostasis. Studies have found that macrophages engulf asbestos and cause its ferroptosis, which increases catalytic iron content inside and outside the cell. Furthermore, this ferroptosis promotes the development of mesothelial carcinoma by activating the Wnt/β‐catenin pathway.^10^ In addition, studies have shown that macrophages can induce the ferroptotic cascade through erythrophagocytosis. A similar pro‐ferroptosis effect can also occur during treatment with ferric citrate or radiation‐induced hemorrhage.^79^, ^80^, ^81^

The expression level of inducible nitric oxide synthase in M1 macrophages is higher than that of M2 macrophages, so more nitric oxide is produced (thus inhibiting lipid peroxidation), and the ferroptosis of M1 macrophages is inhibited.^82^ This study showed that nitric oxide plays an important role in regulating the ferroptosis of macrophages, and it supports a potential therapeutic opportunity for inhibiting M2 macrophages through ferroptosis to enhance the antitumor immune response.

## 5 FERROPTOSIS IN CANCER THERAPY

### 5.1 Targeting ferroptosis

**FIGURE 3:**
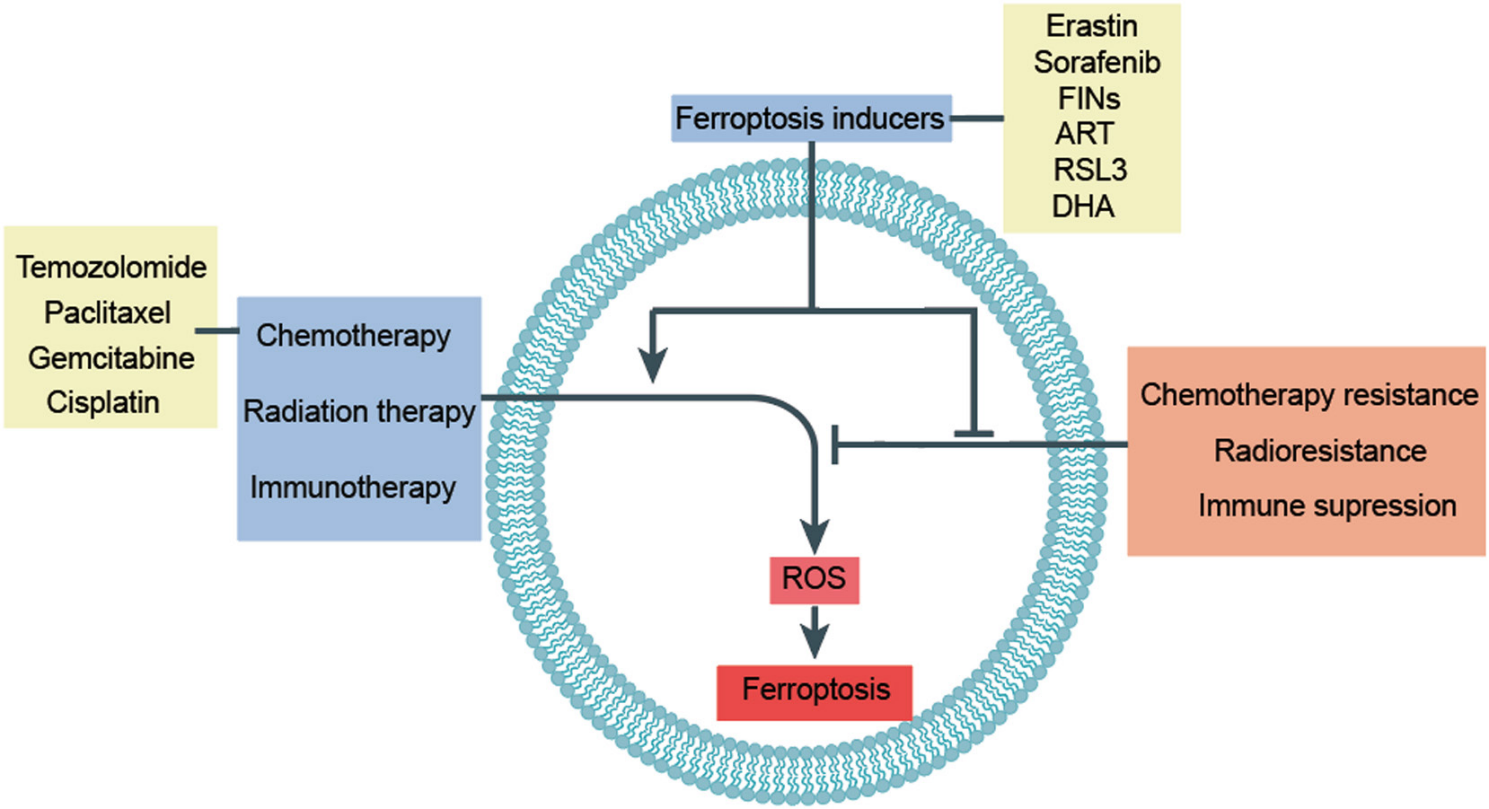
Roles of ferroptosis in antitumor therapy. Three common antitumor treatment methods—chemotherapy, radiotherapy, and immunotherapy—have played important roles in antitumor treatment. However, when they face tumor resistance, radiotherapy resistance, and immunosuppression, they are often ineffective. Ferroptosis has introduced some new methods. These three common treatment methods can promote the ferroptosis of tumor cells together with ferroptosis inducers. Conversely, ferroptosis inducers can enhance these three treatments by reversing tumor resistance, radiotherapy resistance, and immunosuppression.

Killing tumor cells by activating regulatory cell death is an effective anticancer treatment. Although some results have been achieved in clinical cancer treatment, drug resistance of tumor cells remains a troublesome problem.^83^, ^84^, ^85^ The discovery of ferroptosis may improve the clinical treatment of tumors, and exploring the activation of ferroptosis may provide new therapeutic targets^3^ (Figure 3).

Studies have shown that certain refractory tumor cells are abnormally sensitive to ferroptosis.^86^, ^87^ For example, traditional therapies induce apoptosis in cancer cells but are generally ineffective in cancer cells in a mesenchymal state. The enhanced synthesis of PUFA‐PL in these cancer cells makes them strongly dependent on GPX4 to detoxify lipid peroxides for survival. Therefore, these cells are highly susceptible to ferroptosis.^87^ For example, certain recurrent breast cancer cells with mesenchymal features exhibit a high sensitivity to ferroptosis.^88^ Likewise, mesenchymal gastric cancer cells overexpressing very long‐chain fatty acid protein 5 (ELOVL5), a key enzyme involved in PUFA synthesis, are particularly susceptible to ferroptosis.^89^ In addition, subpopulations of dedifferentiated melanoma cells may also be susceptible to ferroptosis due to low levels of GSH.^87^ CD44‐overexpressing mesenchymal cancer cells may increase sensitivity to ferroptosis by increasing intracellular iron load.^90^ In conclusion, cancer cells prone to ferroptosis tend to have the following metabolic characteristics, including high levels of PUFAs, overloaded iron pools, and vulnerable GPX4–GSH defenses. These types of patients may gain more benefits from targeting ferroptosis.

Convincing evidence has shown that activation of the ferroptosis of tumor cells is a promising new method for cancer treatment. In theory, small molecules that can induce ferroptosis can be found through systematic screening or by inhibiting key molecules related to ferroptosis resistance, such as GPX4 and cystine/glutamate reverse transporter SLC7A11, to achieve ferroptosis activation. We review here some ways to make progress in cancer treatment by activating ferroptosis.

Systematic screening identified some small molecules, such as erastin,^3^ sorafenib,^91^ artemisinin (ART),^92^ dihydroartemisinin (DHA),^93^ RSL3, and ferritin‐inducing compounds (FINs), that induced cell death through ferroptosis. For example, in tumor cells that are resistant to sorafenib, the combination of erastin and sorafenib has exerted a powerful antitumor effect.^94^ For example, ART can induce ROS and ferroptosis in pancreatic ductal adenocarcinoma (PDAC) cell lines. Ferroptosis inhibitors can block lipid peroxidation and cell death induced by ART, thereby increasing cell survival.^92^ Studies have shown that DHA specifically promoted cell death in head and neck cancer (HNC) cells by inducing ferroptosis and apoptosis.^93^ In addition, 10 different ART derivatives not only killed tumor cells by inducing apoptosis, autophagy, or necroptosis (as previously established) but also killed tumor cells by ferroptosis.^95^ A larger study discovered that a group of small molecules named FINs can induce ferroptosis.^96^ FIN56 has not only promoted the reduction of GPX4 protein levels but also prevented the production of lipophilic antioxidants, which ultimately triggered the ferroptosis of cells.^96^ However, this method still has great potential to screen more novel ferroptosis inducers.

GPX4 is the cause of ferroptosis induced by lipid peroxidation signals; therefore, knocking down the expression of GPX4 is enough to induce ferroptosis,^17^ and GPX4 inhibitors may be potential candidates for tumor treatment. Because RSL3 effectively inhibits GPX4, identifying GPX4 inhibitors using the structure of RSL3 is a current research interest. For example, a recent study found that covalently targeting a binding site on GPX4 can induce cell ferroptosis.^97^ Ideally, the co‐crystal structure of GPX4 and RSL3 will prompt research on GPX4 inhibitors for therapeutic use. By inhibiting the key active site of GPX4 selenocysteine and systematically screening ferroptosis inducers, it may be possible to combat tumor chemotherapy resistance. However, we should take into account that GPX4 is an essential protein for life, and knocking out GPX4 greatly increased lethality in mouse embryos.^98^ Compared with normal cells, though, cancer cells are more sensitive to GPX4 inhibition. Therefore, treating tumors by inhibiting GPX4 may be a potential treatment method, although the side effects caused by the lack of this key enzyme should be considered in future studies.

To date, study on the safety and effectiveness of ferroptosis‐related drugs is limited *in vivo* and mainly involves PE, (1S, 3R)‐RSL3, and sorafenib. The structure of PE is similar to that of erastin, which is more soluble in water and more stable and suitable for experiments in vivo.^17^ In a xenograft murine tumor model, PE has induced ferroptosis to inhibit tumor growth. In addition, when retinoblastoma protein is reduced, sorafenib can effectively eliminate tumors in murine xenograft models of HCC.^99^ 2‐nitroimidazoles can induce ferroptosis of glioma stem cells under hypoxic conditions.^100^ Solanine can induce ferroptosis of liver cancer cells by inhibiting GPX4 and GSH to increase the level of intracellular ROS.^101^ However, evaluation of drug safety has not been involved in these studies and must be considered in the future.

Because the state of Nrf2 greatly affects the therapeutic response of HCC cells to ferroptosis‐targeted therapy,^91^ the expression of Nrf2 must be suppressed to enhance antitumor efficacy during ferroptosis‐targeted therapy. Furthermore, the iron‐rich tumor environment and the clinical application of sorafenib in the treatment of RCCs indicate that targeting ferroptosis in RCC may be a treatment option.^102^, ^103^ In addition, recent studies have found that autophagy can cause ferroptosis by reducing the level of ferritin in tumor cells.^104^ Overexpression of NCOA4 has always increased the degradation of ferritin and promoted ferroptosis.^104^

Previous studies have shown that inhibition of the cystine/glutamate antiporter SLC7A11 can significantly reverse the resistance of HNC cells to cisplatin, whereas the deficiency of SLC7A11 will not affect the development and survival of mice^103^; compared with chemotherapy, this inhibition may have better efficacy and fewer side effects in antitumor treatments that target ferroptosis. However, inducing ferroptosis by targeting system Xc‐ may not be an effective treatment for certain tumors that do not rely on system Xc‐. Recent studies have found that tumor cells that are sensitive to erastin‐induced ferroptosis are resistant because they lack cysteinyl‐tRNA synthetase.^105^ The lack of cysteinyl‐tRNA synthetase increases the accumulation of cystathionine, which activates the trans‐sulfurization pathway and ultimately makes tumor cells resistant to ferroptosis.^105^ However, the sensitivity of tumor cells to erastin can be restored by inhibiting the trans‐sulfide pathway. In addition, some tumors, such as diffuse large B‐cell lymphoma, not only rely on system Xc‐ but also have defective trans‐sulfurization pathways. Inhibiting system Xc‐ to induce ferroptotic cancer cells may be an effective therapeutic target.^17^ Therefore, the genetic composition of tumor cells determines to a large extent whether antitumor therapy can be carried out by targeting system Xc‐. In general, targeting important molecules in the ferroptosis pathway to treat tumors remains an important direction of exploration for tumor‐targeted therapy.

However, cancer cells can acquire resistance to ferroptosis through different escape mechanisms. Therefore, avoiding cancer cell resistance to ferroptosis is critical to targeting ferroptosis. For example, reducing DJ1 inhibits the activity of S‐adenosyl homocysteine hydrolase (SAHH) and thus the transsulfuration pathway, which resensitizes xenograft tumors to targeting ferroptosis.^106^ Similarly, targeting other enzymes involved in the trans‐sulfuration pathway, such as cystathionine β‐synthase (CBS) and glycine N‐methyltransferase (GNMT), can also enhance tumor sensitivity to ferroptosis^107^, ^108^ . Furthermore, inhibition of pyruvate dehydrogenase kinase 4 (PDK4) by accelerating pyruvate oxidation can resensitize xenograft tumors to ferroptosis‐inducing agents.^109^ Tumor cell resistance to ferroptosis can also be reversed by targeting oncogene‐induced downstream effectors. For example, PI3K can promote tumor cell resistance to ferroptosis through mTOR complex 1 (mTORC1). Thus, the combination of mTORC1 inhibitors and ferroptosis inducers exerted a powerful tumor‐suppressor effect.^110^ Overall, blocking the escape pathways of cancer cells to avoid cancer cell resistance to ferroptosis is an important strategy for cancer therapy (Figure 3).

Because different cancers have different metabolisms, their susceptibility to ferroptosis is different. But, it has not been possible to determine which cancer is more suitable for ferroptosis‐related treatment in the future. However, after reviewing the extensive literature, we found that diffuse large B‐cell lymphoma and renal cell carcinoma are particularly sensitive to GPX4‐mediated ferroptosis^17^ and adrenocortical carcinoma is very sensitive to DDT‐mediated ferroptosis.^111^ Among all cancers, alterations in iron metabolism genes were most abundant in clear cell renal cell carcinoma, and the mRNA levels of 38 iron metabolism genes were most significantly associated with prognosis in clear cell renal cell carcinoma.^112^ In addition, a large number of studies on the mechanism of ferroptosis sensitivity of tumor cells have provided many new targets for targeted ferroptosis therapy, such as lung cancer^113^, ^114^, ^115^, ^116^ and colorectal cancer.^117^, ^118^, ^119^, ^120^ This may indicate that these tumors are more suitable for ferroptosis‐related therapy.

### 5.2 Chemotherapy

Studies have shown that erastin and cisplatin synergistically inhibit the growth of ovarian cancer cells, and inhibition may be manipulated by a mechanism mediated by ROS, which enhances cisplatin therapy and provides a new strategy for overcoming cisplatin resistance.^121^ A recent study showed that classic chemotherapeutic drugs can also induce ferroptosis in cancer cells, which is a new mechanism by which chemotherapeutic drugs exert their effects. For example, cisplatin can reduce the level of GSH in cancer cells to inhibit the effect of GPX4 and ultimately induce ferroptosis of cancer cells^122^ (Table 1). Furthermore, when free iron levels increase as a result of ferritinophagy, cisplatin can trigger ferroptosis.^123^ More evidence has shown that ferroptosis inducers, such as erastin, can synergistically promote the anticancer effect of cisplatin by antagonizing system Xc‐ or GPX4 in a variety of cancers.^122^, ^123^, ^124^, ^125^ In addition, octanoylanilide hydroxamic acid (SAHA), as a histone deacetylase inhibitor, can induce tumor cells to produce ROS to make them sensitive to the effects of cisplatin.^126^

**TABLE 1:** Antitumor therapy together with ferroptosis inducers

Tumor type	Ferroptosis inducer	Combination	Target	Mechanism	Ref.
Acute myeloid leukemia	Erastin	Cytarabine and doxorubicin /adriamycin	GPX4	JNK and p38 synergistically promote erastin‐induced ferroptosis	[140]
Breast cancer	Albiziabioside A	Dichloroacetate	GPX4	AlbA‐DCA can inhibit GPX4 and eliminate M2 macrophages to promote antitumor immunity	[156]
Breast cancer	Ferroptosis inducers	Oxygen‐boosted PDT	–	PDT promotes the accumulation of lymphocytes at the tumor site and stimulates the secretion of IFN‐γ	[158]
Glioblastomas	Sulfasalazine	Gamma knife radiosurgery	System Xc‐	SSZ inhibits the uptake of cystine and therefore reduces the level of GSH, thus increasing the intracellular ROS	[149]
Head and neck cancer	Trigonelline	Artesunate	NRF2	NRF2 inhibitor trigonelline can induce lipid peroxide accumulation	[134]
Lung cancer	RSL3	Cisplatin	GPX4	RSL3 enhances the therapeutic effect of cisplatin	[123]
Melanoma	TGF‐β inhibitor	PD‐1 antibody	ROS	PD‐1 antibody and TGF‐β inhibitor cooperatively polarize M2‐TAMs into M1‐TAMs and promote the Fenton reaction with Fe ions discharged from magnetic nanoclusters	[155]
NSCLC	Erastin	Cisplatin	GSH‐GPX4	Cisplatin can deplete the GSH and inactivate the GPX4 together	[122]
NSCLC	Erastin	Cisplatin	ROS	SAHA and erastin, the inducers of ROS‐mediated cell death, strongly enhanced the effect of cisplatin in WT EGFR cells	[126]
Ovarian cancer	Erastin	Cisplatin	System Xc‐	Erastin can inhibit system Xc‐ and potentiate the cytotoxic effects of cisplatin to eradicate tumor cells	[125]
PDAC	Erastin	Gemcitabine	System Xc‐	Both SLC7A11‐KO cell lines exhibit amino acid stress with induction of ATF4 and cell death	[124]
PDAC	Erastin	Cisplatin	System Xc‐	Both SLC7A11‐KO cell lines exhibit amino acid stress with induction of ATF4 and cell death	[124]
Sarcoma	Anti–PD‐L1/Anti‐ CTLA‐4 mAb	Ionizing radiation	System Xc‐	Radiation therapy and interferon synergistically reduce the expression level of SLC7A11	[151]

The ferroptosis caused by ferritinophagy is related to the antitumor mechanism of ART and its derivatives.^93^, ^127^, ^128^ Ferritin binds to NCOA4 and is transported to the lysosome. However, ART and its derivative dihydroartemisinin (DAT) increase the degradation of ferritin in the lysosome, which ultimately increases intracellular iron and triggers ferroptosis.^129^, ^130^ In addition, recent literature has shown that DAT can regulate iron homeostasis by changing the ratio of iron regulatory proteins/iron response elements and so increase the level of free iron in tumor cells.^127^ In this manner, the synergistic treatment of ART and transferrin induced ferroptosis in pancreatic ductal adenocarcinoma by promoting iron transport into cells and accelerating the release of free iron from lysosomes.^92^

Traditional chemotherapeutics can upregulate GPX4 and system Xc‐ to inhibit cancer cell death and ferroptosis. For example, gemcitabine can increase the expression level of heat shock protein 70 family protein 5 (HSPA5), thereby preventing the degradation of the GPX4 protein and ferroptosis in cancer cells.^131^ However, it is possible to prevent the protective effect of gemcitabine on tumors and promote the eradication of pancreatic cancer by combining treatment with erastin or inhibiting the HSPA5‐GPX4 pathway^124^ (Table 1). Temozolomide (TMZ) promoted the expression of SLC7A11 at the gene and protein levels through the Nrf2 and ATF4 pathways. In addition, TMZ can enhance the activity of cystathionine γ‐lyase (CTH); when SLC7A11 was blocked, CTH was an important enzyme that ensured the supply of cysteine and the synthesis of GSH in the trans‐sulfide pathway. This finding may explain the mechanism by which gliomas with high system Xc‐ expression levels are more likely to be treated by the combination of erastin and TMZ.^132^, ^133^ Artesunate can be used for chemotherapy of HNC cells, but it can activate the Nrf2 antioxidant pathway. Therefore, inhibition of Nrf2 can induce lipid oxidation of HNC and reverse its ferroptosis resistance.^134^

Other classic chemotherapy drugs enhance their anticancer effects by combining with ferroptosis inducers. Paclitaxel (PTX), a classic chemotherapy drug widely used to treat a variety of cancers, can not only upregulate the expression of p53 and p21 genes but also downregulate the expression of SLC7A11 and SLC1A5.^135^, ^136^ A recent study has shown that the combined use of PTX and RSL3 can cause the ferroptosis of cancer cells.^137^ Natália et al.^138^ found that metformin can induce ferroptosis in breast cancer cells by reducing the level of intracellular GSH. In addition, MAP30, a biologically active protein isolated from balsam pear seeds, can increase the therapeutic effect of cisplatin on ovarian cancer by inducing cell ferroptosis.^139^ Similarly, erastin can significantly enhance the inhibitory effects of cytarabine and adriamycin on leukemia cells. Its mode of action has nothing to do with RAS and partly depends on the induction of ferroptosis. The c‐Jun N‐terminal kinases (JNKs)/p38‐MAPK pathway is an important cause of erastin‐induced leukemia cell death^140^ (Table 1). In addition, the ERK‐MAPK pathway plays a major role in RAS‐dependent ferroptotic cells.^141^ Fingolimod, a new type of immunosuppressant, also induces ferroptosis and autophagy through the PP2A/AMPK pathway.^142^ These findings suggest that promotion of erastin as an anticancer treatment *via* activation of the MAPK pathway may be a promising approach.

### 5.3 Radiation therapy

The ionizing radiation of radiotherapy always damages cells by inducing DNA double‐strand breaks.^143^ In addition, the activation of oxidases and the radiolysis of water will indirectly lead to GSH depletion and increased ROS.^144^, ^145^, ^146^ Studies have shown that depletion of glutathione promotes the antitumor effect of radiotherapy.^147^, ^148^ The expression of SLC7A11 in glioblastoma or glioma cells is higher than that in normal brain tissue. Therefore, inhibition of system Xc‐ by ferroptosis inducers can enhance the radiotherapy effect of glioblastoma.^149^ Khorsandi et al.^150^ proved that synergistic treatment with pre‐irradiation and gallic acid could greatly inhibit the survival of tumor cells, mainly by inhibiting GPX4.

Recently, Lei et al.^150^ demonstrated that in most cancer cell lines, ionizing radiation (IR) promotes the expression of ferroptosis genes, but it also induces ROS through the ACSL4/LPCAT3/ALOX and SLC7A11/GPX4 pathways. In addition, inhibition of SLC7A11 or GPX4 can increase the radiosensitivity of tumor cells to IR.^150^ Ferroptosis may play an important role in combined radiotherapy and CD8^+^ T cell therapy. Studies have shown that the ataxia telangiectasia mutant, which is upregulated by radiotherapy, can synergize with interferon (IFN) to downregulate the expression of SLC7A11.^151^ A recent study found that itraconazole can induce the ferroptosis of tumor cells by increasing the level of iron in the lysosome and thus can reverse the resistance of nasopharyngeal carcinoma stem cells to radiotherapy.^152^ These findings provide new directions for improving the therapeutic effect of radiotherapy or regulating ferroptosis to treat radiotherapy‐resistant tumors (Figure 3).

### 5.4 Immunotherapy

Recent studies have shown that cytotoxic CD8^+^ T cells can suppress the expression level of system Xc‐ by secreting IFN‐γ and thus promote the ferroptosis of cancer cells.^153^ This finding suggests that the combination therapy of immunotherapy and a ferroptosis inducer may be a promising direction for research. PD‐1 antibody treatment can transform M2 macrophages into M1 macrophages, enhancing the antitumor function of macrophages.^154^ Nanoparticles are composed of PD‐1 antibodies and transforming growth factor beta inhibitors, which synergistically enhance the antitumor immune response, increasing H_2_O_2_ levels in M1 macrophages and inducing a Fenton reaction. The subsequent generation of hydroxyl radicals induces ferroptosis in tumor cells.^155^ A new drug, AlbA‐DCA, inhibits the progression of breast cancer by downregulating the expression of GPX4 and eliminating precancerous M2 TAMs^156^ (Table 1).

M1 phagocytes have a higher resistance to ferroptosis than M2 phagocytes do.^82^ Studies have shown that radiated tumor cell–released microparticles (RT‐MPs) can transform M2 macrophages into M1 macrophages.^157^ In addition, RT‐MPs can also promote the ferroptosis of tumor cells and trigger ICD, which improves the clearance of tumor cells by macrophages.^154^ A recent study found that PDT can effectively induce ferroptosis and phenotypic maturation of bone marrow mesenchymal stem cells in cancer cells.^50^ The combination of PDT and ferroptosis inducers can reduce the level of GSH in tumor tissues and increase the level of the lipid peroxidation product malondialdehyde. In addition, PDT promoted not only the infiltration of lymphocytes at the tumor site but also the secretion of IFN‐γ.^158^ Therefore, the combination of PDT and immunotherapy to promote the ferroptosis of tumor cells and exert anti‐tumor effects requires more exploration.

## 6 PERSPECTIVE

Since the discovery of ferroptosis, continual evidence has suggested inextricable links between ferroptosis and tumor immunity. Many studies have suggested that the immunogenicity of ferroptotic cells will increase. For example, two immunogenic markers, HMGB1 and CRT, were increased in ferroptotic cells.^4^, ^46^ In addition, ferroptosis can directly affect the functions of various immune cells, suggesting that the function of immune cells can be regulated by targeting ferroptosis to treat cancer. Therefore, exploration of the interaction mechanism between ferroptosis and immunity can provide ideas for new antitumor treatments.

Targeting metabolic signatures of ferroptosis, such as enrichment of PUFA‐PLs, iron overload, and unbalanced ferroptosis defense system, have uncovered a series of novel cancer therapeutic targets. The drugs developed with these targets can not only be used for antitumor therapy alone but also can be combined with conventional therapy that can induce apoptosis, which can further improve the therapeutic effect. For example, the resistance of tumor cells to chemotherapy and radiotherapy has been a major problem in antitumor therapy. However, the emergence of ferroptosis offers hope for a solution to this problem. The combination of cisplatin and ferroptosis inducers restored sensitivity to cells that were originally cisplatin‐resistant. In addition, some other refractory tumors, such as radio resistant and immunosuppressive types, can also be greatly improved by ferroptosis inducers.

Finally, in order to realize the full potential of ferroptosis in tumor treatment strategies, there are some additional issues that need to be addressed urgently in future studies. Thorough histological and pharmacological analysis must be performed to confirm whether ferroptosis inducers have potentially toxic effects on normal tissues. In addition, it is critical to discover predictive biomarkers that can predict tumor response to ferroptosis‐inducing therapy, especially those that can be detected directly in patient fluids and biopsy specimens. These biomarkers help stratify tumor patients for further antitumor treatment with ferroptosis‐inducing therapy. In conclusion, both further exploration of the mechanism of ferroptosis and the development of new drugs are expected to provide more help for antitumor therapy.

## AUTHOR CONTRIBUTIONS

**Chuandong Gong:** Writing – original draft (lead). **Qiankun Ji:** Writing – original draft (equal). **Miaojing Wu:** Writing – original draft (equal). **Zewei Tu:** Writing – review and editing (supporting). **Kunjian Lei:** Writing – review and editing (supporting). **Min Luo:** Writing – review and editing (supporting). **Junzhe Liu:** Writing – review and editing (supporting). **Li Lin:** Writing – review and editing (supporting). **Kuangxun Li:** Writing – review and editing (supporting). **Jingying Li:** Project administration (equal). **Kai Huang:** Project administration (equal). **Xingen Zhu:** Project administration (lead).

## FUNDING INFORMATION

This research was funded by the National Natural Science Foundation (grant nos. 82002660, 81960456, 81760445, and 81760446), the Key Research and Development projects in Jiangxi (grant nos. 20212BBG73021), Jiangxi Training Program for academic and technical leaders of major disciplines – Young talents program (grant no. 20212BCJ23023), and Jiangxi Province Department of Education Science and technology research project (grant no. GJJ210177).

## CONFLICT OF INTEREST

No competing interests exist.

## Data Availability

Data sharing is not applicable to this article as no new data were created or analyzed in this study.
